# On prioritising global health’s triple crisis of sepsis, COVID-19 and antimicrobial resistance: a mixed-methods study from Malawi

**DOI:** 10.1186/s12913-022-08007-0

**Published:** 2022-05-07

**Authors:** Paul Kawale, Levi Kalitsilo, Jessie Mphande, Bayode Romeo Adegbite, Martin P. Grobusch, Shevin T. Jacob, Jamie Rylance, Nyovani J. Madise

**Affiliations:** 1 African Institute for Development Policy, Lilongwe, Malawi; 2 Centre de Recherches Médicales de Lambaréné (CERMEL) and African Partner Institution, Lambarene, Gabon; 3 Centre of Tropical Medicine and Travel Medicine, Department of Infectious Diseases, Amsterdam Infection & Immunity, Amsterdam University Medical Centres, Amsterdam Public Health, University of Amsterdam, location AMC, Amsterdam, The Netherlands; 4 Institut für Tropenmedizin, Universität Tübingen, Tübingen, Germany; 5 Institute of Infectious Diseases and Molecular Medicine, University of Cape Town, Cape Town, South Africa; 6 Masanga Medical Research Unit, Masanga, Sierra Leone; 7 Liverpool School of Tropical Medicine, Liverpool, UK; 8 Walimu, Uganda; 9 Malawi-Liverpool-Welcome Trust, Blantyre, Malawi

**Keywords:** Sepsis, Health system, Health priority, Antimicrobial resistance, COVID-19

## Abstract

Sepsis causes 20% of global deaths, particularly among children and vulnerable populations living in developing countries. This study investigated how sepsis is prioritised in Malawi’s health system to inform health policy. In this mixed-methods study, twenty multisectoral stakeholders were qualitatively interviewed and asked to quantitatively rate the likelihood of sepsis-related medium-term policy outcomes being realised. Respondents indicated that sepsis is not prioritised in Malawi due to a lack of local sepsis-related evidence and policies. However, they highlighted strong linkages between sepsis and maternal health, antimicrobial resistance and COVID-19, which are already existing national priorities, and offers opportunities for sepsis researchers as policy entrepreneurs. To address the burden of sepsis, we recommend that funding should be channelled to the generation of local evidence, evidence uptake, procurement of resources and treatment of sepsis cases, development of appropriate indicators for sepsis, adherence to infection prevention and control measures, and antimicrobial stewardship.

**Supplementary Information:**

The online version contains supplementary material available at 10.1186/s12913-022-08007-0.

## Introduction

Sepsis is a common complication of infection that frequently results in death or serious disability. In the 2020 report on the global epidemiology and burden of sepsis, the World Health Organization (WHO) estimated that this life-threatening condition occurs in fifty million people each year, and is responsible for eleven million deaths (1 in 5 of all deaths globally) [1]. Of those, at least 2 million sepsis deaths are estimated to occur in Africa [2]. This is likely to be a significant underestimate, as sepsis in African countries is under-recognised, derived from limited diagnostic capability, variable reporting, and the lack of universal registrations of deaths [3].

All populations can be affected by sepsis. Children are most frequently affected, including three million newborn babies and 1.2 million older children every year [4]. Sepsis is the third-leading cause of maternal death with over 95% of maternal sepsis deaths occurring in developing countries [5, 6]. Non-pregnant adult populations are also at risk [7], particularly those who are immuno-suppressed, including people living with the HIV virus [8].

Amid the global coronavirus pandemic, emerging evidence warns that the most serious complications of COVID-19 include sepsis [9–13]; and COVID-19 reinforces the need to improve sepsis care [13]. With the emergence of COVID-19, epidemics such as Ebola, Marburg virus disease, Lassa fever, malaria, dengue and measles have also re-emerged in several Low- and Middle-Income Countries (LMICs) [7, 14–17]. The treatment of these epidemics has been challenging for health systems due to the similarity in the presentation of the disease to COVID-19, thereby making misdiagnosis likely. Meanwhile, over three-quarters of COVID-19 patients are treated with antibiotics [18], and antibiotics are also the most common treatment for sepsis [19]. This risks possible inappropriate use of antibiotics, leading to antimicrobial resistance (AMR) [20].

Most cases of sepsis are treated in hospitals, where deaths are more common among men than women [21], and the condition accounts for 40% of mortality in Malawi’s intensive care units [22]. Overall, in-hospital case-fatality rates reach as high as 48% in Malawi, with neonatal aetiologies driven by group *B* Streptococcus (GBS), *Streptococcus pneumoniae* and non-typhoidal Salmonella (NTS) [23, 24], with Enterobacteriaceae and other gram-negative bacteria also important in older people. Resource limitations mean that only one-third of Surviving Sepsis Campaign (SSC) recommendations [25] can be implemented in Malawi’s health facilities [26]. Furthermore, knowledge of these guidelines amongst Malawian medical students and health care workers is limited [27]. Beyond Malawi, other countries in Africa are facing similar challenges in their health systems because of inadequate resources such as beds and health care workers– which fall 60% below the UN’s least limit [17, 28, 29].

**Figure 1.**
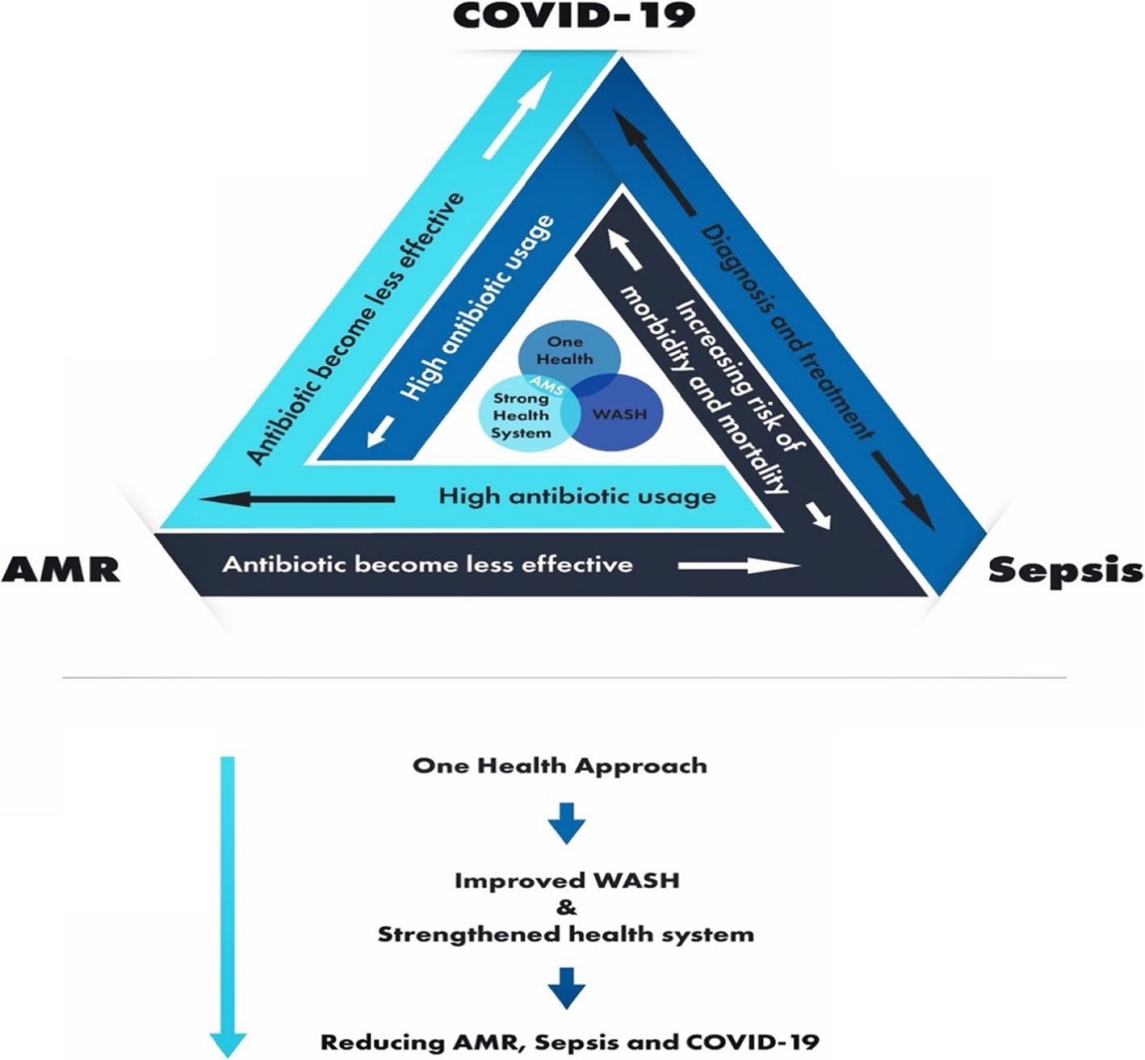
Relationship between sepsis, AMR and COVID-19 ( Source: www.afidep.org)

While the high prevalence of sepsis and COVID-19 demand for more antibiotic usage to address them, the growing AMR calls for limited use of antibiotics to reduce AMR spread. While this appears to present a competing situation, there exist some positive relationships. Linkages are seen on how the three (Sepsis, COVID-19 and AMR) occur and are addressed collectively through a One Health approach [30]; antimicrobial stewardship (AMS), a robust health system, improving hygiene in the health facilities, and improving water, sanitation and hygiene (WASH) in the communities, as conceptualised in Figure 1 below.

On the contrary, there are no similar integrated disease management approaches that have been adopted to prevent and manage re-emerging epidemics thereby causing a delay in the treatment as the health care workforce has been focused on containing the COVID-19 pandemic [7]This is worsened by the minimal financing that many LMICs allocate to the health sector. COVID-19’s impact on the management of other epidemics has been very evident. Due to the COVID-19 pandemic, previous management strategies such as for Ebola in the Democratic Republic of Congo were also ceased and Ebola response teams were redirected to COVID-19 response [14].

Effective treatment and management of epidemics requires robust and strong disease surveillance systems but these are underdeveloped in LMICs. Often, all efforts are redirected to the most current epidemic thereby making tracing of other infectious diseases problematic which can contribute to disease spread and misdiagnosis and control of suspected cases [7, 31].

This study is part of the policy engagement component of the African Research Collaboration on Sepsis (ARCS), a multinational research initiative funded by the UK National Institute for Health Research (NIHR) which aims to: 1) deliver high-quality sepsis research training; 2) establish commonly agreed sepsis care quality indicators which could form the bedrock of programme monitoring and evaluation and 3) pilot-test innovative sepsis care interventions. This component of the project aimed to describe baseline perceptions on the prioritisation of sepsis among key stakeholders in Malawi in the context of a health system disrupted by COVID-19 and to identify opportunities for ongoing strategic stakeholder engagement. This broad remit requires sustainable partnerships between government policymakers and parliamentarians; policy influencers including researchers, civil society organisations, and the media; and policy implementers (healthcare workers, and non-government organisations). To be successful, these stakeholders need to share a common understanding of sepsis, and the evidence which informs actions to address it [32].

Our study, which focuses on the policy landscape that shapes the management and control of sepsis, AMR and COVID-19, strengthens the knowledge base on the linkages between these three issues and their impact on Malawi’s health system, with broader lessons for other LMICs with similar healthcare systems.

## Methods

### Study site and stakeholders

This study was conducted in Malawi in the three main cities of Mzuzu, Lilongwe and Blantyre to yield broad sub-national and national-level stakeholder voices. Stakeholders included policymakers, policy influencers and policy implementers.

### Stakeholder involvement

Stakeholder involvement was ensured throughout the study process. Research/training institutions and MOH were first involved at the conceptualisation of the research as part of the ARCS programme. Research questions and outcome measures were developed and informed by stakeholder priorities, experience and preferences, captured at the 2019 ARCS Annual Workshop in Dar es Salam, Tanzania, using the RAPID Outcome Mapping Approach (ROMA) [33]). During the design phase, researchers reviewed the background literature and methodology of the study.

### Recruitment of study participants

During participant recruitment for the study, letters were sent to heads of 31 purposively identified institutions comprised of 8 stakeholder groups in Malawi’s health sector, asking them to each nominate a key informant. Criteria used for inclusion in the study were: staff aged 18 years or older, having heard of sepsis, English speaking and willing to provide informed consent for the interview. Those whose role was purely administrative were excluded from the study. Twenty-five institutions from 6 stakeholder groups each provided a key informant. Participants were provided with information about the study to help them to give informed consent. After reading the study information sheet, study participants accepted to be interviewed and audio-recorded by signing a consent form.

### Data collection

A qualitative semi-structured questionnaire was used in face-to-face interviews and voice calls which were conducted in English. The questionnaire was adapted from the sample Bellwether tool developed by Coffman and Reed [34]. This questionnaire asks study participants about their perceptions of policy agenda priorities; their perceptions of the characteristics and capacities of sepsis-related policy stakeholders, both advocates and opponents; and factors that might raise sepsis on the policy agenda. These key informant interviews were audio recorded with permission, detailed notes were taken and transcribed.

Study participants also completed a quantitative tool to rate the likelihood of sepsis-related policy outcomes being realised in the next five years, on a scale of 1-5 (where: 1 = highly unlikely; 2 = unlikely; 3 = neither likely nor unlikely; 4 = likely and 5 = highly likely). Participants were asked to recall what MOH policymakers are saying about sepsis; consider what language the policymakers are using; judge how interested and open the policymakers are to the topic of sepsis and suggest what kind of evidence will convince them. For outcomes relating to policy-influencing stakeholders, participants took into consideration who is engaging in sepsis and how influential they are; what can be done to involve others; what the influential stakeholders are saying about sepsis; and what new legislation, budgets, programmes or strategies are being developed that can relate to sepsis. For outcomes related to policy implementers, participants considered who is involved in implementing sepsis-related policies; whether they have the skills, relationships and incentives to deliver; whether different stakeholders are working coherently together to implement sepsis-related policy; and whether the necessary structures and incentives are in place to facilitate this.

### Data analysis

Quantitative data were analysed to come up with the respondents’ perception of likely sepsis-related policy outcomes. For instance, they were asked about the likelihood of policymakers in the Ministry of Health to demand for sepsis evidence. Respondents then rated the likelihood from 1 (highly unlikely) to 5 (highly likely). All responses were entered into a spreadsheet and modes of each outcome were aggregated and compared against stakeholder type and participant demographics.

Qualitative questions were asked in pre-identified areas for discussion and analysis. We entered key informant interview transcripts into a spreadsheet. With the aid of NVivo, we employed word cloud analysis, a text-matching process of identifying and locating particular text matches in raw data [35]. Framework Analysis [36] was also employed for the qualitative data through five phases: familiarisation, identifying a thematic framework, indexing, charting, and mapping and interpretation [37]. During the thematic framework identification phase, we used the interview guide in a deductive process of identifying broad themes, then another deductive categorisation using Kingdon’s multiple streams model for policy influence [38]. This thematic framework was refined inductively by identifying emerging themes or issues from the data.

## Results

### Characteristics of study participants

Interviews were completed with 20 of 22 identified key informants (Table 1). These represented six stakeholder groups comprising policymakers (three directorates in the Ministry of Health), policy influencers (two research/training institutions, three media houses, two civil society organisations) and policy implementers (three central hospitals, three ethics/regulatory bodies). Of note, we did not have representation from the private sector. There were more male (60%) than female study participants. The majority of the study participants (*N =*17) were aged over 31 years, with 65% of them having a postgraduate degree. The distribution of participants by profession is shown in Table 1.

**Table 1:** Type and Number of Study Participants

**Profession**	**Medical Doctor**	**Researcher**	**Journalist**	**Economist**	**Nurse**	**Public Health Specialist**	**Laboratory Technician**	**Total**
**Stakeholder Group**	**7**	**1**	**3**	**1**	**3**	**4**	**1**	**20**
Ministry of Health	1			1		1		3
Ethics/Regulatory Body	1				1	1		3
Research/Training Institution		1			1			2
Central Hospital	5				1		1	7
Civil Society Organisation						2		2
Media			3					3
**Age Group**								**20**
26 – 30	2					1		3
31 – 40	1		2	1		2	1	7
41 +	4	1	1		3	1		10
**Gender**								**20**
Male	5	1	1	1		3	1	12
Female	2		2		3	1		8
**Highest Qualification**								**20**
Undergraduate degree/ MBBS	1		1			2	1	5
Postgraduate degree	6		2	1	2	2		13
Doctorate		1			1			2

### Likely sepsis policy outcomes

In our quantitative survey, study participants estimated the likelihood of sepsis-related policy outcomes being realised within the next five years among policy influencers, policymakers and policy implementers, as shown in Table 2. For policy influencers, participants estimated that training institutions were highly likely to agree on a definition of sepsis. They also suggested that ethics committees and regulatory bodies were highly likely to recognise sepsis as a priority and grant permission for researchers to audit patients’ clinical records. Researchers will then more likely supply the resultant sepsis evidence to policymakers. Our study participants felt that policymakers at MOH are highly likely to use sepsis as an indicator of the Malawi health system’s quality. As policy implementers, health workers from central hospitals and members of health worker unions were thought highly likely to participate in sepsis training. These results are explored further in our qualitative findings below.

**Table 2:** Participants’ Estimation of the Likelihood of Sepsis-Related Outcomes, Rated from 1 (‘Not likely at all’) to 5 (‘Most likely’), (*N =*13)

**Stakeholder Group**	**Outcome**	**Likelihood of Outcome (Mode)**
MOH	Demand evidence on sepsis	4
	Organise training workshops on sepsis for health workers	4
	Put sepsis as the indicator of the quality of the health system	5
Central hospitals & CSO (health worker unions)	Participate in training workshops on sepsis	5
	Accurately diagnose and report sepsis	4
	Recognition of sepsis as a priority disease	4
Ethics committees & Regulatory bodies	Give permissions for clinical audit of patient records for quality improvement	5
	More multi-disciplinary clinical research on sepsis	4
	Recognition of sepsis as a priority disease	5
Research organisations	Supply evidence on sepsis	5
	Implement policy engagement activities on sepsis	4
	Organise conference tracks on sepsis	4
Training institutions	Agree on a definition of sepsis	5
	Teach how to accurately diagnose and report sepsis	4
	Future health workers recognise sepsis as a priority disease	4
International funders	Convene stakeholders’ meetings on sepsis	4
	Put out calls for proposals around sepsis	3
	Put sepsis on international donors' agenda	3
Media	Disseminate evidence on sepsis	3
	Implement public engagement activities on sepsis	3
	Commemorate World Sepsis Day	2
Health workers	Participate in training workshops on sepsis	5
	Accurately diagnose and report sepsis	4
	Recognition of sepsis as a priority disease	4

### Malawi health policy agenda priorities

**Figure 2.**
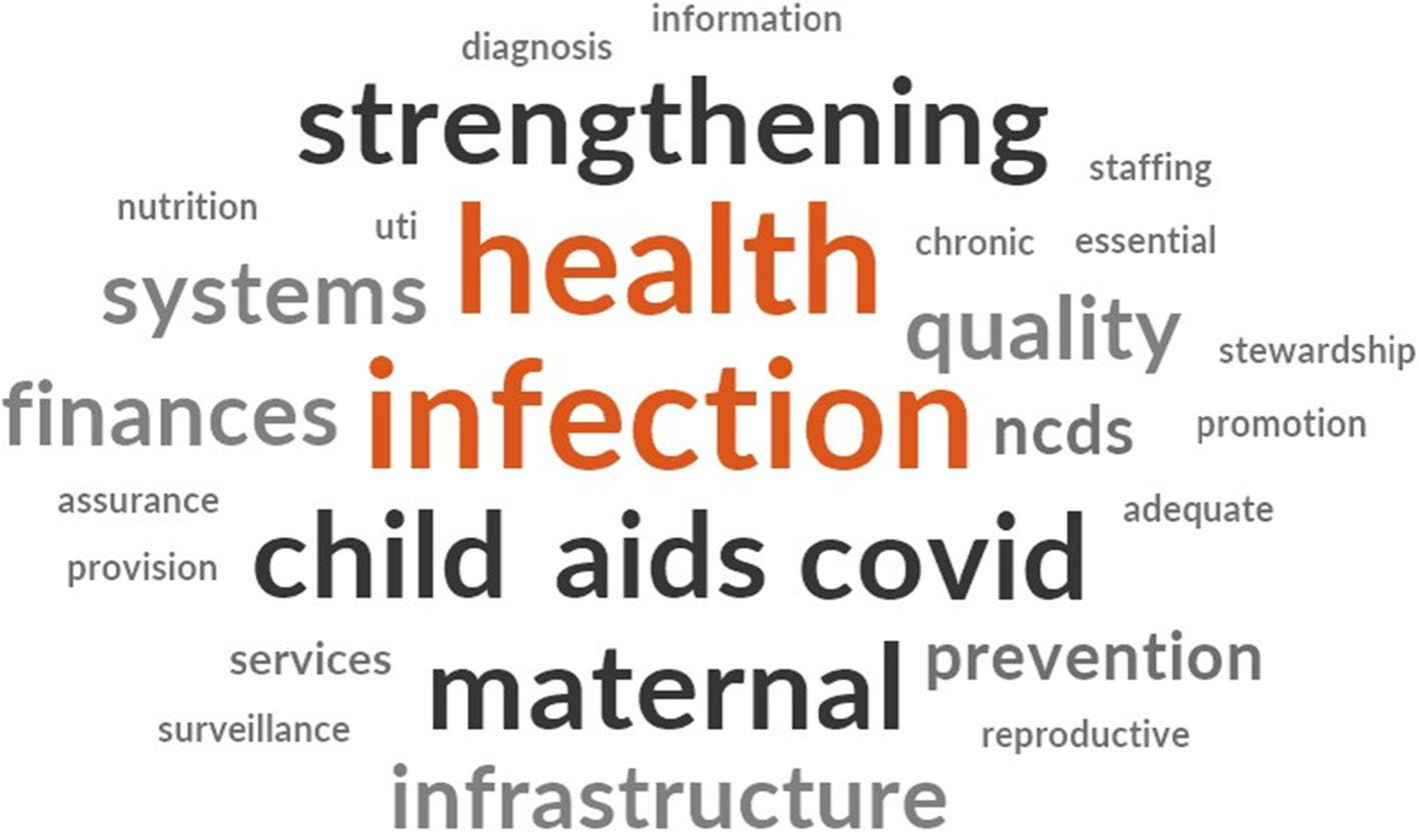
Respondents' Perceptions of Malawi Health Sector Priorities

In our qualitative interviews, we asked participants what the top three priorities are for the Ministry of Health. Figure 2 shows the word cloud of participants’ responses. Most respondents mentioned health system strengthening priorities (provision of quality adequate essential services, staffing, finances, information, infrastructure); health promotion priorities for prevention, diagnosis and surveillance of infectious diseases (COVID-19, urinary tract infection [UTI], AIDS, antimicrobial stewardship); and maternal and child health-related priorities (nutrition, reproductive health, chronic non-communicable diseases [NCDs]).

From the Framework Analysis, our study respondents additionally mentioned universal health coverage as one of the MOH’s priorities. Only a laboratory technician and a medical doctor, both at central hospitals, mentioned sepsis as a priority. Details of perceived health sector priorities by study participants’ occupation, stakeholder group, age, gender and qualification are in Supplementary File 1.

### Perceived characteristics and capacities of sepsis-related stakeholders

We asked study participants to mention stakeholders involved in sepsis-related policies and service delivery as both advocates and opponents, and whether they have the skills, relationships and incentives to deliver.

#### Policy influencers

Our respondents cited patients, survivors of sepsis, health managers and health workers (critical care, internal medicine and surgery) as the most appropriate advocates for sepsis policy. Many of our respondents also estimated the likelihood of state regulatory bodies to recognise sepsis as a priority disease to be highly likely (rated 5), particularly because of its links with COVID-19.

*“As of now we have COVID-19 which can trigger sepsis with the so many remedies that people are trying without evidence. So, it is urgent. We may learn later that most patients dying from COVID-19 could be because of sepsis … We do not need another year … We need to learn from how COVID-19 has been devastating. Next year may be too late.”* – Male medical doctor

Most respondents estimated that it is highly likely (rated 5) for training institutions to agree on a definition of sepsis, and likely (rated 4) for them to teach how to accurately diagnose and report sepsis.

Despite the media being an active agent of advocacy, they were perceived to be weak supporters of anti-sepsis campaigns. The media was perceived with mostly only an estimated likelihood of neither likely nor unlikely (rated 3), neither likely nor unlikely (rated 3) and unlikely (rated 2) to disseminate evidence on sepsis, implement public engagement activities on sepsis, and commemorate World Sepsis Day, respectively. This was noted to be illustrated by the lack of sepsis awareness among the public and media, as explained by this journalist:

*“If a disease is subtle in the way it kills people, not so many will be aware of it. There is a need for more awareness, therefore, so that people are aware of its magnitude and start treating it as seriously as it should. People are pointing at another direction away from sepsis on causes of mortality because they are not aware of it.”* – Male journalist

The respondents identified opponents to sepsis as traditional healers, traditional birth attendants (TBA) and religious groups. They observed that traditional healers and TBAs are viewed as opponents since they usually do not adhere to required infection prevention and control (IPC) measures when discharging their duties, thus promoting sepsis cases in their patients. Religious leaders were also viewed as opponents of fighting sepsis due to their strong opposition to safe abortions.

#### Policymakers

Our respondents stated that the MOH has highlighted the quality of care as a priority to improve infection prevention, led by the Reproductive Health Directorate and the Quality Management Directorate. Most respondents indicated that it is highly likely (rated 5) for MOH to put sepsis as the indicator of the quality of Malawi’s health system.

However, most respondents were not aware of a sepsis policy that includes prevention, treatment and rehabilitation, as exemplified by a research/training institution key informant below. Notwithstanding this, most participants estimated that it is likely (rated 4) for MOH to demand evidence on sepsis.

*“We are doing this as islands. Queen Elizabeth [Central Hospital in Blantyre] are doing what they think they are supposed to do. It will not be very different from what Kamuzu Central Hospital is doing, but then we don’t have a policy to my knowledge*.” –Male researcher

#### Policy implementers

Most respondents recognised that Malawian health workers have the skills to implement policies and provide sepsis care at their individual levels, with most respondents estimating that it is likely (rated 4) for health workers to recognise sepsis as a priority disease. As a result, most respondents estimated that, in the medium term, it is likely (rated 4) for health workers to accurately diagnose and report sepsis. However, many respondents reported that health workers use clinical judgement for diagnosis, as illustrated by a research/training institution key informant below. This is because they were not aware of any clear guidelines and indicators for reporting the various types of sepsis in Malawi.

*“[On the] clinical side is not easy to diagnose sepsis. Sometimes they mistake sepsis for malaria. Policymakers have problems as well because of the nature of sepsis.” –* Female research nurse

Our respondents also observed that health workers’ heavy workload and limited skills often lead to them making poor decisions when it comes to prescriptions. Within hospitals, some respondents bemoaned poor communication amongst health workers, which negatively affects the delivery of high-quality services. They particularly described a lack of communication between the laboratory, the pharmacy and the clinical area as leading to inadequate quality of sepsis care.

Antimicrobial resistance (AMR) is high on the MOH’s agenda, according to our respondents. In particular, respondents expressed how the Pharmacy and Medicines Regulatory Authority does not adequately regulate private pharmacies that dispense antibiotics without laboratory tests, which leads to AMR and may also delay the patient from seeking appropriate sepsis care. They mentioned a government programme on antibiotic stewardship. They recommended that the programme’s priority should be regulating the selling of antibiotics in private pharmacies if antimicrobial stewardship is to be taken seriously. Participants reported that even the strongest antibiotics were no longer effective:

*“If we are looking at the strong antibiotic which we have been using is ceftriaxone. This is our bazooka as of now. But you discover that because of this willy nilly giving of antibiotics, this bazooka is also showing resistance.”* – Male medical doctor

Study participants also identified oversight and accountability structures that can improve sepsis prevention and care as not functional at most health facilities due to busy schedules and lack of information sharing, as exemplified by a public health key informant below. These include ‘Quality Improvement Surveillance Teams’, ‘Antimicrobial Stewardship Committees’, ‘Infection Prevention Focal Persons’ and ‘Quality Improvement Focal Persons’.

*“You may discover some people may just move from one meeting to another and instead of let’s say, at a facility, instead of taking one person to learn, at times most of the times those people don’t have the time to disseminate.”* – Female public health specialist

To overcome these obstacles, they described a best practice of using social media for electronic patient referrals in Malawi’s southern region:

*“Like in the south, we have one [WhatsApp] group that encompasses all districts in the southern region. The districts will refer patients electronically and this [is] open to everybody and then the specialists can make comments, guide them on how to manage or may recommend that the patients be referred to a referral centre.”* – Male medical doctor

### Factors that might raise sepsis on the policy agenda

Our third objective aimed at establishing factors that might raise sepsis on the policy agenda. In trying to establish this, participants were asked about any important issues that needed to be promoted or developed to effect sepsis policy (new legislation, budgets, programmes or strategies). Participants identified information and advocacy, resource allocation (finance and medical commodities) and maternal health issues as core to putting sepsis on the policy agenda.

#### Information and advocacy

Study respondents described the inadequacy of routine health management information system (HMIS) data to provide information on sepsis due to inadequate indicators for sepsis and mislabelling and under-reporting of sepsis data. Researchers in our study recognised this gap and spoke of the need to include sepsis indicators in HMIS, such as the total number of suspected sepsis cases and the total number of laboratory-confirmed sepsis cases, to gather evidence for sepsis advocacy to policymakers.

Our study participants advised that sepsis advocates will need clinical and laboratory evidence on the prevalence of sepsis, its causes in Malawi, its pathways to mortality, and its contribution to maternal, HIV and non-communicable disease deaths, as elucidated by a key informant below.

*“More diseases are linked to sepsis, even the current COVID-19 … We have common killers in Malawi now and what we need is to show the relationship between those diseases and sepsis. So, if we say people with AIDS when they get an infection they end up with septic shock and death. People with maternal issues, people involved in traffic accidents will develop an infection, later on, sepsis and septic shock … So, measures must be taken to prevent sepsis arising from the many diseases … I think we stand a better chance of getting resources and we stand a better chance of appealing to the policymakers to start thinking about sepsis.”* – Male public health specialist

Respondents in our study also stated that for policy issues to attract finance ministers and research funders, there is a need for advocacy on how sepsis affects the health system. They noted that, currently, there is insufficient information to convince policymakers and funders on sepsis. Therefore, the study respondents recommended advocating to the Directorate of Clinical Services to lead the development of national policy and guidelines.

*“Normally, we have indicators that we use to track from the District [Health] Information System, DHIS 2. There are a number of indicators. So, these are normally like, we need to review them and appraise based on need. So, if the evidence is generated and then we see how big of a problem it is, for sure I don’t see any reason why it shouldn’t be prioritized.” –* Male economist.

In comparison, most of our respondents also estimated that it is likely (rated 4) for more multi-disciplinary clinical research on sepsis to be conducted, with Malawi’s research institutions highly likely (rated 5) to supply that evidence. These research institutions were estimated by most respondents to likely (rated 4) implement policy engagement activities on sepsis.

#### Resource allocation

Respondents noted the difficulty in distributing resources from domestic revenue among the different government sectors. Nevertheless, they noted that the government prioritises the health sector; however, most of the priority is perceived by our respondents to be on preventive health, and they reported finding it difficult to counter-argue for continued investment in curative critical care to prevent sepsis deaths, as illustrated by a central hospital key informant quoted below. Our respondents further reported that the resources remaining for curative services do not support all the needed services. They mentioned the laboratory as the service least supported, and clinical services as the most supported, despite new funders now supporting laboratory services.

*“People in public health have varied arguments. Their argument is we must focus on primary health care; it is cheaper and then with little resources you are able to make a bigger impact. Those of us who work in the intensive care unit, while we agree with that argument, we still think although it is expensive to treat sepsis in intensive care, et cetera, we still need to invest in this.”* – Female medical doctor

According to our respondents, most funders are not interested in sepsis. They estimated that development partners in Malawi are ambivalent (neither likely nor unlikely (rated 3)) about putting out calls for sepsis research, and as well as ambivalent about putting sepsis on international donors' agenda. Respondents gave an example of COVID-19, which has over-shadowed development partners’ financing of sepsis-related services:

*“So, we are side-lining other important infections including sepsis. It’s hard now to justify that maybe you buy us blood culture bottles, which are very expensive, of course. Because if you go there and say we are running out of test kits maybe for COVID-19, it’s easy to get their assistance than these other things … It’s now silent.”* – Male economist

Respondents also observed that supplies of sepsis-related drugs and other medical equipment, for instance, diagnostic equipment, were not quickly replenished at health facilities. Availability of such supplies would as well address issues around sepsis care and management. One reason given by the respondents for this situation was the Central Medical Stores Trust’s (CMST) restrictions on local procurement by cost centres, even while CMST does not have the capacity to deliver:

*“They don’t have and they will say, ‘No, our policies say, or maybe the procurement processes say, you should only buy from other supplies maybe 10 million in a month. Like materials worth 10 million and not more than that.’ And yet what Central Medical Stores [doesn’t] have is a lot more than that amount given.”* – Female nurse

Within this context, respondents suggested that the government be more concerned about the availability of sepsis-related drugs and supplies in health facilities, and ensure that women are being cared for in clean health facilities:

*“The availability of supply, I mean availability of sterile gloves when you are doing vaginal examinations. Availability of, what’s this, antiseptic solution. Availability of prophylaxis and antibiotics. The availability of antibiotics themselves. Clean water. Yeah. Clean theatre environment.”* – Female medical doctor

#### Maternal health

Respondents also reported that maternal health is high on the MOH agenda. They said that, although women in Malawi die or become infertile because of puerperal sepsis that results from post-partum infections, evidence for this is difficult to obtain. Respondents in our study reported that women delivering at home or with traditional birth attendants contribute to sepsis cases in Malawi. Our respondents said there is a strong possibility that such women would deliver in very unhygienic conditions, exposing them to infections that would result in sepsis, as narrated by a male public health specialist:

*“But you still find that people in the communities will still go to traditional birth attendants or traditional healers for things like unsafe abortions, for deliveries. And [with] whatever happens, most of these cases will have for example sepsis and related things.”*

Furthermore, respondents acknowledged that this risk is also present at hospitals due to nosocomial infections, and recommended stronger reproductive health policies that will protect women in delivery wards from infections. Participants noted that promoting better quality maternal health services would as well promote sepsis policy. They acknowledged that measures to prevent infection are not adhered to in many health facilities due to lack of safe running water, use of the same linen before and after an operation in the theatre, and congestion:

*“Congestion is another contributing factor. You find that if a hospital, for example, Bwaila is congested you [are] doing 16 Caesarean sections maybe only in the night. You don’t have enough time to clean up the place for the next patient.”* – Male medical doctor

## Discussion

We found three broad themes in our study describing how sepsis is prioritised in Malawi. These themes are sepsis’ mixed level of priority in Malawi; maternal health as a flagship for sepsis prioritisation; and linking sepsis with AMR and COVID-19. We discuss these themes in the context of the global health scientific discourse.

### Sepsis: a mixed level of priority in Malawi

Our study has found that sepsis is perceived to not be a priority among Malawi’s policymakers, funders and the media. Other studies equally highlight that huge resource implications associated with sepsis care and the non-harmonised definition contribute to delayed prioritisation in many health systems [39–42]. WHO has declared sepsis a global health priority [43]. However, critical care resource allocation to manage sepsis is low in both high- and low-income countries [44–46]. This indicates that regions and countries do not necessarily adopt the same priorities as global health institutions. Other studies recognise that policymakers only begin to consider an issue as a problem if there has been a major event that has drawn attention to it; if there are external actors pushing for the adoption of policy or even elections [39, 40]. This could be due to a lack of local evidence to justify the adoption of global problems into the regional and country context.

On the other hand, we found that sepsis is perceived to be a priority by Malawi’s health research institutions due to its effect on maternal health. Previous studies have similarly found that sepsis is prioritised by research institutions [31, 41, 42]. Researchers can play an active role as policy entrepreneurs who can provide evidence to for highlighting the problem of sepsis, develop evidence to support policies documents, and using windows of opportunity to engage policymakers [39]. Despite this, insufficient knowledge translation and policy engagement by the research institutions limits the local evidence available to Malawi’s policymakers and influencers. As a result, policymakers make decisions in an environment of uncertainty [39]. This limits how empirical evidence can guide public policy and, as a result, limits the potential of public policies to improve the well-being of societies [43].

This inadequate availability of local sepsis evidence is compounded by a lack of localised guidelines and inadequate HMIS data. This is further aggravated by Malawi not having a standard definition of sepsis, leading to misdiagnosis and inaccurate recording. Sepsis is further often masked by underlying health conditions [1, 34, 44], making it difficult to diagnose and accurately report it in HMIS records to provide the needed evidence for policymakers. More research therefore would help in understanding sepsis leading to a common definition based on empirical evidence.

### Maternal health as a flagship for sepsis prioritisation

Our study has revealed that quality of care and maternal health are perceived to be MOH priorities in Malawi. Evidence suggests that maternal sepsis is a growing problem in both developed and developing countries [1, 45, 46]. In Malawi, between 16.3% and 29.4% of maternal mortality is attributed to sepsis [47] thereby requiring improved service provision and early identification of an illness. This is significant because we also found that nosocomial infections are considered by Malawi’s stakeholders as the key causes of maternal sepsis, similar to other developing countries [48, 49].

There is a need for global and national policymakers to develop standardised maternal sepsis management guidelines. These will benefit from current innovations in obstetric early warning systems [50], thereby improving both the quality of care as well as prevention of sepsis caused by nosocomial infections. The guidelines will enable policymakers, service providers, patients and their carers to prioritise maternal sepsis to make appropriate, life-saving decisions.

In Malawi’s Ministry of Health, quality of care and maternal health priorities are led by the Quality Management Directorate and Reproductive Health Directorate, respectively. Nonetheless, some respondents explained that these priorities are not translating into improved service delivery, with infection prevention measures not being adhered to in the country’s health facilities because of inadequate oversight and accountability structures. In other similar settings, although hospital staff may have the knowledge of good quality improvement practices, adherence to these practices is low, as observed through lack of disinfection, low handwashing and poor waste management [51–53]. Strengthening health facilities’ infection prevention oversight and accountability structures will require behavioural and systems thinking approaches.

### Linking sepsis with AMR and COVID-19 priorities

We also found that AMR and COVID-19 are high on Malawi’s MOH policy agenda. This is significant because unregulated prescription and supply of antibiotics were found in our study to further compound the problem of sepsis. The development of AMR poses a challenge and tension when treating sepsis patients [20]. Where sepsis is more promptly recognised, and where healthcare workers have a high degree of suspicion of the diagnosis, the corollary is an increased use of antimicrobials and inevitably emerging drug resistance to first-line antibiotics such as amoxicillin, chloramphenicol and co-trimoxazole [52]. When identified early and treated timely with antibiotics, sepsis fatality risk is reduced, but it usually results in high antibiotic usage [8, 47, 54, 55], leading to AMR, which is now a serious global public health problem.

At the service delivery level, our study found this increased use of antibiotics to be partly caused by limited resources and communication between the laboratory, the pharmacy and the clinical area. Other studies have also recognised the communication problems among departments in health facilities that lead to late detection of sepsis and delayed prescription of appropriate antibiotics [56, 57]. AMR is growing, pulling things in the opposite direction by increasing the mortality risk for sepsis through a reduction in the probability of cure with initially appropriate antibiotic therapy [58]. AMR is therefore a major factor determining clinical unresponsiveness to treatment and rapid evolution to sepsis and septic shock [59].

At the policy level, possible causes of medical commodity shortages were identified as Central Medical Stores Trust’s restrictions on hospitals’ procurement from private suppliers, and inadequate regulation of private pharmacies selling antibiotics. Studies done in Malawi and other developing countries have demonstrated the lack of regulated sale of antibiotics in private pharmacies, which has massive implications on antimicrobial stewardship (AMS), with one study revealing that up to 95% of private medicine stores sold amoxicillin without prescription, although it is a prescription-only medicine in Malawi [60, 61].

On the other hand, both sepsis and COVID-19 share clinical characteristics such as multi-organ failure and T and NK cell exhaustion [62]. Currently, sepsis with subsequent multi-organ dysfunction is one of the main causes of death in COVID-19 patients [63]. Since the basic principles of management of COVID-19 patients are very similar to any kind of viral sepsis, it has been seen that some management protocols for critically ill COVID-19 patients are based on experience with bacterial sepsis [64]. In some cases, severe COVID-19 is viewed as sepsis induced by viral infection [65].

The excessive use of antibiotics has also been observed in treating patients presenting with cough and fever, which can be symptoms of bacterial community-acquired pneumonia. Treatment using antibiotics is also common where there is difficulty in ruling out bacterial co-infections [66, 67]. A review of cases shows that the proportion of patients diagnosed with COVID-19 and bacterial co-infections was only 6.9% [66], while over 74% of patients with COVID-19 are treated with antibiotics [18]. COVID-19 has also brought the limitations of acute medical care in Africa into sharp focus [13], but how countries across the continent responded to it also demonstrated that early recognition and careful evidence-based care for critically ill patients can drastically reduce mortality.

There are limitations in our study that need to be taken into consideration when interpreting the findings. First, respondents were drawn from three cities in Malawi at central hospitals and MOH headquarters. This left out district-level policymakers, who are growing in policy influence as Malawi becomes more decentralised. Furthermore, although there was co-creation in identifying stakeholders, there are usual shifts in players in policy spaces, particularly during the 2019 and 2020 elections that saw a new government formed in Malawi. We attempted to overcome this limitation by conducting interviews over 18 months. Similarly, the sepsis policy outcomes in our quantitative tool may have shifted during this period. Being a baseline study, such changes should be identified during subsequent assessments.

## Conclusion and recommendations

Despite WHO setting sepsis as a global health priority, our study illustrates that there are often mismatches between global priorities and priorities of countries such as Malawi. This is particularly when the country’s funders and public (through the media) do not prioritise the issue. To contextualise global sepsis priorities into local health systems, there is a need for local evidence, uptake of evidence and political will.

To achieve this, we recommend that global health donors adequately fund WHO priorities based on available global evidence, such as the sepsis incidence and mortality analysis from the Global Burden of Disease Study [68]. This funding needs to be channelled to treatment of sepsis, generation of local sepsis evidence and improving evidence uptake by policymakers. Evidence should focus on linkages between sepsis and the country’s existing health sector priorities, such as maternal health, infectious diseases and health system strengthening [17]. Research uptake should be through stakeholder engagement, particularly the media, and the generation of strategic communications products from research, such as animation, infographics and policy briefs. This would therefore lead stakeholders to draw on their emotions to challenge beliefs about the nature of sepsis problems and appropriate solutions [39].

We also recommend that the Ministry of Health use this evidence to put appropriate sepsis indicators in the HMIS and build the capacity of health workers to accurately identify and report sepsis. Evidence on the magnitude of sepsis can capture the attention of policymakers and be used to make a persuasive case for anti-sepsis policy. This should enable MOH to develop appropriate sepsis guidelines for both sepsis management and prevention, such as those to improve communication among health workers providing sepsis-related services (e.g., clinical services, laboratory, pharmacy). This will see HMIS data on sepsis used as performance and quality indicators for health facilities and the health system, similar to other settings [69].

To reduce hospital-acquired infections which include sepsis, COVID-19 and the spread of AMR, while reducing healthcare costs, [7, 14, 15]we recommend the development of behavioural and system interventions that promote maternal health care workers’ adherence to set infection prevention measures that clearly define sepsis.

To prevent the spread of superinfections, and ensure that sepsis and COVID-19 are treatable in the future, we recommend reserving antibiotics for severe bacterial infections, ensuring microbiological testing capabilities, and adhering to best prescribing practices. Capacity building of hospitals in Malawi is essential to adequately procure medical supplies from local vendors to supplement the Central Medical Stores Trust’s limited capacity. We similarly recommend strict restrictions on the sale of antibiotics in local private pharmacies, and investments in public awareness of AMR. Further, we recommend additional studies on the relationship between sepsis, AMR and COVID-19 to refine our conceptual framework.

## Supplementary Information

**Additional file 1.**

## Data Availability

The data used and analysed during this study are available from the corresponding author on reasonable request.

## Abbreviations

AMR: Antimicrobial Resistance
AMS: Antimicrobial Stewardship
ARCS: African Research Collaboration on Sepsis
CSO: Civil Society Organisation
GBS: Group B Streptococcus
HMIS: Health Management Information System
MOH: Ministry of Health
NIHR: National Institute for Health Research
NTS: Non-Typhoidal Salmonella
ROMA: RAPID Outcome Mapping Approach
SSC: Surviving Sepsis Campaign
TBA: Traditional Birth Attendants
TH: Traditional Healers
WASH: Water, Sanitation and Hygiene

## References

[1] Rudd KE, Johnson SC, Agesa KM, Shackelford KA, Tsoi D, Kievlan DR (2020). Global, regional, and national sepsis incidence and mortality, 1990–2017: analysis for the Global Burden of Disease Study. The Lancet..

[2] World Health Organization. WHO Sepsis Technical Expert Meeting. WHO. 2018. https://www.who.int/servicedeliverysafety/areas/sepsis_meeting2018/en/. Accessed 17 Sep 2019.

[3] Lewis JM, Abouyannis M, Katha G, Nyirenda M, Chatsika G, Feasey NA (2019). Population incidence and mortality of sepsis in an urban African setting 2013–2016. Clinical Infectious Diseases..

[4] Fleischmann-Struzek C, Goldfarb DM, Schlattmann P, Schlapbach LJ, Reinhart K, Kissoon N (2018). The global burden of paediatric and neonatal sepsis: a systematic review. The Lancet Respiratory Medicine..

[5] Black RE, Laxminarayan R, Temmerman M, Walker N. Reproductive, Maternal, Newborn, and Child Health. The International Bank for Reconstruction and Development / The World Bank; 2016. 27227235

[6] Say L, Chou D, Gemmill A, Tunçalp Ö, Moller AB, Daniels J (2014). Global causes of maternal death: A WHO systematic analysis. The Lancet Global Health..

[7] Okonji OC, Okonji EF, Mohanan P, Babar MS, Saleem A, Khawaja UA (2022). Marburg virus disease outbreak amidst COVID-19 in the Republic of Guinea: A point of contention for the fragile health system?. Clinical Epidemiology and Global Health..

[8] Waitt PI, Mukaka M, Goodson P, SimuKonda FD, Waitt CJ, Feasey N (2015). Sepsis carries a high mortality among hospitalised adults in Malawi in the eraofantiretroviral therapy scale-up: A longitudinal cohort study. Journal of Infection..

[9] Cascella M, Rajnik M, Cuomo A, Dulebohn SC, di Napoli R. Features, evaluation and treatment coronavirus (COVID-19). In: StatPearls [Internet]. Treasure Island (FL): StatPearls Publishing; 2020. 32150360

[10] Ryan DH, Ravussin E, Heymsfield S (2020). COVID 19 and the Patient with Obesity-The Editors Speak Out. Obesity..

[11] Little P (2020). Non-steroidal anti-inflammatory drugs and covid-19. BMJ..

[12] Zhou F, Yu T, Du R, Fan G, Liu Y, Liu Z (2020). Clinical course and risk factors for mortality of adult inpatients with COVID-19 in Wuhan, China: a retrospective cohort study. The Lancet..

[13] Nsutebu E, Rylance J, Appiah JA, Grobusch MP, Williams G, Kissoon N (2021). COVID-19 reinforces the need to improve sepsis care resources in Africa. Infection..

[14] Khan FMA, Hasan MM, Kazmi Z, dos Santos Costa AC, Aborode AT, Ahmad S (2021). Ebola and COVID-19 in Democratic Republic of Congo: grappling with two plagues at once. Tropical Medicine and Health..

[15] Costa AC dos S, Hasan MM, Xenophontos E, Mohanan P, Bassey EE, Hashim HT (2021). COVID-19 and Zika: An emerging dilemma for Brazil. J Med Virol.

[16] Yousaf A, Khan FMA, Hasan MM, Ullah I, Bardhan M (2021). Dengue, measles, and COVID-19: A threefold challenge to public health security in Pakistan. Ethics, Medicine and Public Health..

[17] Mohanan P, Islam Z, Hasan MM, Adedeji OJ, dos Santos Costa AC, Aborode AT (2022). Malaria and COVID-19: A double battle for Burundi. African Journal of Emergency Medicine..

[18] Cox MJ, Loman N, Bogaert D, O’Grady J (2020). Co-infections: potentially lethal and unexplored in COVID-19. The Lancet Microbe..

[19] Dondorp AM, Limmathurotsakul D, Ashley EA (2018). What’s wrong in the control of antimicrobial resistance in critically ill patients from low- and middle-income countries?. Intensive Care Medicine..

[20] Busch LM, Kadri SS (2020). Antimicrobial Treatment Duration in Sepsis and Serious Infections. J Infect Dis..

[21] Waitt P, Downie P, Mukaka M, Toh CH, Heyderman R (2009). Sepsis: Markers of Mortality in Malawi. Journal of Infection..

[22] Gundo R, Lengu ES, Maluwa A, Mtalimanja O, Chipeta D, Kadyaudzu C (2014). An Audit of Admissions to Intensive Care Unit at Kamuzu Central Hospital in Malawi. Open Journal of Nursing..

[23] Milledge J, Calis JCJ, Graham SM, Phiri A, Wilson LK, Soko D (2005). Aetiology of neonatal sepsis in Blantyre, Malawi: 1996–2001. Annals of Tropical Paediatrics..

[24] Krajčinović SS, Doronjski A, Barišić N, Stojanović V (2015). Risk factors for neonatal sepsis and method for reduction of blood culture contamination. Malawi Medical Journal..

[25] Levy MM, Evans LE, Rhodes A (2018). The Surviving Sepsis Campaign Bundle: 2018 update. Intensive Care Medicine..

[26] Abdu M, Wilson A, Mhango C, Taki F, Coomarasamy A, Lissauer D (2018). Resource availability for the management of maternal sepsis in Malawi, other low-income countries, and lower-middle-income countries. International Journal of Gynecology and Obstetrics..

[27] Marshall-Brown P, Namboya F, Pollach G (2016). Evaluating sepsis training for medical students and nonphysicians in Malawi. Journal of Clinical Anesthesia..

[28] Ghosh S, Moledina N, Hasan MM, Jain S, Ghosh A, Colossal challenges to healthcare workers combating the second wave of coronavirus disease,  (2019). (COVID-19) in India. Infection Control & Hospital Epidemiology..

[29] Hasan MM, Costa AC dos S, Xenophontos E, Mohanan P, Bassey EE, Ahmad S (2021). Lassa fever and COVID-19 in Africa: A double crisis on the fragile health system. Jo Med Virol.

[30] Ahmad T, Hui J (2020). One Health approach and Coronavirus Disease 2019. Human Vaccines & Immunotherapeutics..

[31] Aborode AT, Hasan MM, Jain S, Okereke M, Adedeji OJ, Karra-Aly A (2021). Impact of poor disease surveillance system on COVID-19 response in africa: Time to rethink and rebuilt. Clinical Epidemiology and Global Health..

[32] Silberberg B, Aston S, Boztepe S, Jacob S, Rylance J (2020). Recommendations for fluid management of adults with sepsis in sub-Saharan Africa: a systematic review of guidelines. Critical Care..

[33] Young J, Shaxson L, Jones H, Hearn S, Datta A, Cassidy C. RAPID outcome mapping approach: a guide to policy engagement and influence. London: Overseas Development Institute; 2014.

[34] Coffman J, Reed E, Network I (2009). Unique methods in advocacy evaluation. Retrieved February..

[35] Alqahtani A, Alhakami H, Alsubait T, Baz A (2021). A Survey of Text Matching Techniques. Engineering, Technology & Applied Science Research..

[36] Srivastava A, Thomson SB (2009). Framework Analysis: A Qualitative Methodology for Applied Policy Research. Journal of Administration and Governance..

[37] Green J, Thorogood N (2014). Qualitative Methods for Health Research.

[38] King A, Kingdon JW (1985). Agendas, alternatives, and public policies, Boston: Little, Brown, 1984, xi 240 pp. J Public Policy..

[39] Rylance J, Nsutebu E, Mergani KO, Grobusch MP, Jacob ST (2018). The African Sepsis Alliance: making a difference in the fight against sepsis in Africa. Infection..

[40] Brief P. POLICY BRIEF A CALL TO ACTION : Sepsis is Africa ’ s Neglected Silent Killer. 2019; March.

[41] Dünser MW, Schultz MJ. Arjen M. Dondorp Sepsis Management in Resource-limited Settings. 2019. 32091674

[42] Vincent JL (2016). The Clinical Challenge of Sepsis Identification and Monitoring. PLoS Medicine..

[43] Reinhart K, Daniels R, Kissoon N, Machado FR, Schachter RD, Finfer S (2017). Recognizing Sepsis as a Global Health Priority — A WHO Resolution. New England Journal of Medicine..

[44] Baelani I, Jochberger S, Laimer T, Otieno D, Kabutu J, Wilson I (2011). Availability of critical care resources to treat patients with severe sepsis or septic shock in Africa: A self-reported, continent-wide survey of anaesthesia providers. Critical Care..

[45] Austin S, Murthy S, Wunsch H, Adhikari NKJ, Karir V, Rowan K (2014). Access to urban acute care services in high- vs. middle-income countries: An analysis of seven cities. Inten Care Med.

[46] Mendsaikhan N, Begzjav T, Lundeg G, Brunauer A, Dünser MW (2016). A nationwide census of ICU capacity and admissions in Mongolia. PLoS ONE..

[47] Abdu M, Wilson A, Mhango C, Taki F, Coomarasamy A, Lissauer D (2018). Resource availability for the management of maternal sepsis in Malawi, other low-income countries, and lower-middle-income countries. International Journal of Gynecology & Obstetrics..

[48] Cairney P, Zahariadis N. Multiple streams approach: a flexible metaphor presents an opportunity to operationalize agenda setting processes. Handb Public Policy Agenda Setting. 2016:87–105. 10.4337/9781784715922.00014.

[49] Béland D, Howlett M. The Role and Impact of the Multiple-Streams Approach in Comparative Policy Analysis.

[50] Macalintal J (2016). Moving Evidence into Practice : Early Sepsis Identification and Timely Intervention in the Emergency Department ( Project Code Sepsis ). Intensive Care Medicine..

[51] Scott JT, Larson JC, Buckingham SL, Maton KI, Crowley DM (2019). Bridging the Research-Policy Divide: Pathways to Engagement and Skill Development. American Journal of Orthopsychiatry..

[52] Parfitt SE, Bogat ML, Hering SL, Ottley C, Roth C (2017). Sepsis in obstetrics: clinical features and early warning tools. MCN. Amer J Maternal/Child Nur.

[53] Van Dillen J, Zwart J, Schutte J, Van Roosmalen J (2010). Maternal sepsis: Epidemiology, etiology and outcome. Current Opinion in Infectious Diseases..

[54] Shahida SM, Islam A, Dey BR, Islam F, Venkatesh K, Goodman A (2016). Hospital Acquired Infections in Low and Middle Income Countries: Root Cause Analysis and the Development of Infection Control Practices in Bangladesh. Open Journal of Obstetrics and Gynecology..

[55] Ssekitoleko RT, Oshabaheebwa S, Munabi IG, Tusabe MS, Namayega C, Ngabirano BA (2020). The role of medical equipment in the spread of nosocomial infections: a cross-sectional study in four tertiary public health facilities in Uganda. BMC Public Health..

[56] Edwards W, Dore S, van Schalkwyk J, Armson BA (2020). Prioritizing Maternal Sepsis: National Adoption of an Obstetric Early Warning System to Prevent Morbidity and Mortality. Journal of Obstetrics and Gynaecology Canada..

[57] Sethi T, Awasthi R. Use of artificial intelligence based models for learning better policy for maternal and child health. Eur J Public Health. 2020;30(Supplement_5):ckaa165.291. 10.1093/eurpub/ckaa165.291.

[58] Sarker MAB, Harun-Or-Rashid M, Hirosawa T, Hai MSBA, Siddique MRF, Sakamoto J (2014). Evaluation of knowledge, practices, and possible barriers among healthcare providers regarding medical waste management in Dhaka. Bangladesh. Medical Science Monitor..

[59] Uddin MN, Islam MR, Yesmin K (2014). Knowledge on Hospital Waste Management among Senior Staff Nurses Working in a Selected Medical College Hospital of Bangladesh..

[60] Joshi SC, Diwan V, Tamhankar AJ, Joshi R, Shah H, Sharma M (2012). Qualitative study on perceptions of hand hygiene among hospital staff in a rural teaching hospital in India. Journal of Hospital Infection..

[61] Musicha P, Cornick JE, Bar-Zeev N, French N, Masesa C, Denis B (2017). Trends in antimicrobial resistance in bloodstream infection isolates at a large urban hospital in Malawi (1998–2016): a surveillance study. The Lancet Infectious Diseases..

[62] Seni J, Najjuka CF, Kateete DP, Makobore P, Joloba ML, Kajumbula H (2013). Antimicrobial resistance in hospitalized surgical patients: A silently emerging public health concern in Uganda. BMC Research Notes..

[63] Bin Abdulhak AA, Al Tannir MA, Almansor MA, Almohaya MS, Onazi AS, Marei MA (2011). Non prescribed sale of antibiotics in Riyadh, Saudi Arabia: A Cross Sectional Study. BMC Public Health..

[64] Alp E, Leblebicioglu H, Doganay M, Voss A (2011). Infection control practice in countries with limited resources. Annals of Clinical Microbiology and Antimicrobials..

[65] Drahnak DM, Hravnak M, Ren D, Haines AJ, Tuite P (2016). Scripting nurse communication to improve sepsis care. MEDSURG Nursing..

[66] Ayukekbong JA, Ntemgwa M, Atabe AN (2017). The threat of antimicrobial resistance in developing countries: causes and control strategies. Antimicrobial Resistance & Infection Control..

[67] Machado FR, Cavalcanti AB, Bozza FA, Ferreira EM, Angotti Carrara FS, Sousa JL (2017). The epidemiology of sepsis in Brazilian intensive care units (the Sepsis PREvalence Assessment Database, SPREAD): An observational study. The Lancet Infectious Diseases..

[68] Maki G, Smith I, Paulin S, Kaljee L, Kasambara W, Mlotha J (2020). Feasibility study of the world health organization health care facility-based antimicrobial stewardship toolkit for low-and middle-income countries. Antibiotics..

[69] Chikowe I, Bliese SL, Lucas S, Lieberman M (2018). Amoxicillin quality and selling practices in urban pharmacies and drug stores of Blantyre, Malawi. American Journal of Tropical Medicine and Hygiene..

